# A State Asylum Service: A Change That Would Promote Efficiency

**Published:** 1916-11-25

**Authors:** 


### A STATE ASYLUM SERVICE.

### A Change that would Promote Efficiency.

The *Times*, which is not as well advised in medical matters as it used to be, has recently flown a kite in the direction of a State Medical Service, and the British Medical Association has hastened to protest, and to proclaim its disapproval of the project. After the collapse of the British Medical Association in the matter of the Insurance Act, neither the State, nor the *Times*, nor anyone else is likely to attach any importance to its protest on this or any other subject, and in any case its protest against a State Medical Service is premature, for we do not in the least know what is meant by these three words, and they are susceptible of the most various meanings. They may be held to mean that the State will forbid anyone to practise medicine who has not passed an examination conducted by State officials. There is such a law in Germany, but German methods are not as popular in this country as they were two and a half years ago. The State is busy just now on other matters, and the newspapers are teeming with projects which the State is to take in hand as soon as the war is over. If a tithe of these projects are taken up by the State, it will have its hands so full that it is very unlikely to have time to spare for the institution of a one-portal test for doctors, especially as it does already, through the General Medical Council, secure that the examinations conducted by private licensing bodies shall be satisfactory.

### The Military Model.

Or a State Medical Service may mean that medicine shall in future be a Civil Service, that doctors shall be paid by the State as prison doctors now are, and, like prison doctors, shall be compelled to reside here or there as the Service commands, shall attend such patients as are allotted to them by the Service, and shall pass their time chiefly in keeping official books, filling up official forms, and making out official certificates. The prospect is alluring. The profession would be organised in a hierarchy of official ranks. At the head would be a Physician of State, or a Secretary of State for Medicine, who would, of course, be a barrister or a solicitor, or, if the Conservatives were in office, a wealthy country squire. Subordinate to him would be Under-Secretaries of State, Assistant Under-Secretaries of State, Deputy Assistant Under-Secretaries of State, First-class Physicians, Second-class Physicians, Third-class Physicians, and so forth. Or the Service might be modelled on the Army, and be commanded by Disease Marshals, Physician Generals, Physician Colonels, Physician Majors, Physician Captains, Subalterns, and Privates. The Service would then be put into uniform, with badges of crossed thermometers for the Fever Corps, crossed stethoscopes for the Auscultatory Corps, crossed test-tubes for the Laboratory Corps, and so forth, and the Disease Marshal Commanding-in-Chief would pass his time in devising new buttons and altering the cut of the tunics, so that the Subaltern Physicians should not have too much money to spend on horse-racing and polo. The treatment of disease would be embodied in a series of King's Regulations, and a physician who should presume to treat a patient otherwise than as prescribed in the King's Regulations would be tried by Court Physic, and sentenced to seven days' bandage-rolling or splint-padding.

### State Control of the Asylum Service.

There is one province of medicine that might properly be assumed by the State, and that, if administered by the State, would be benefited rather than harmed. This is the Asylum Service. Lunatic asylums are already under municipal control, and if the control were transferred from the counties and boroughs to the State the service of asylums, though it would suffer in some ways, would probably benefit considerably on balance. At present, except in very large administrative areas, such as London and Lancashire, the administration of each asylum is separate and isolated. Each asylum is administered by a committee, which is not always in session, which meets in many cases not more often than once a month, and in the meantime the asylum is administered by one man, the manager or medical superintendent. The whole efficiency of the management depends in practice, therefore, on the efficiency of the superintendent, and it is of the greatest importance that in choosing this officer the committee should have a wide choice of efficient men, and should have competent means of judging which is the most efficient of the applicants for the post. Under the present system neither of these conditions exists. The applicants are men who have served in subordinate positions in other asylums, and as a rule have occupied that position for many years. This is as it should be, for the administration of an asylum is a very specialised business, and needs a considerable apprenticeship before it can be thoroughly well performed. But, unfortunately, the junior posts in the Asylum Service do not attract the best men. Asylums are for the most part isolated in remote country districts. There is little society, and what there is is not congenial to a man of intellectual tastes. There is no professional emulation, and very little stimulus to scientific work. Promotion depends, not on professional efficiency or eminence, but on administrative ability, and hence professional efficiency is neglected. Promotion is slow and capricious, and depends much less on the merits of the candidate than on the ability and willingness of his chief to write a good testimonial. For these reasons energetic, ambitious, able men look askance at asylum service; seldom enter it; if they do enter it, seldom remain in it; and if they do remain in it, gradually lose their energy and their ambition. Hence the choice of committees when a new superintendent is to be appointed is limited to a class of men that is not the best the profession could furnish, and even out of this class there is no surety that the committee will choose the best or the second best. For in most cases the committee have no personal knowledge of the candidates. They come from distant asylums out of the jurisdiction of the committee, which has to rely on such testimonials and reports as the candidate can produce. It may be that the ablest and best candidate has a chief who does not want to part with him, or, if he does, cannot write a satisfactory testimonial. It may be that a very inferior candidate has a chief who wants to get rid of him, and writes him a glowing testimonial with that purpose in view, a shocking proceeding, no doubt, but there is as much human nature in the staffs of asylums as among other men.

If the Asylum Service were turned into a State Service, as the Prison Service has with the best results been converted, it would attract a better class of man even than the Prison Service, at any rate if the salaries were maintained on the same scale as at present. The higher posts in the Asylum Service are highly remunerated; and if asylums throughout the country were under a single management, the rate of promotion would be regular. All the men throughout the Service would be known at headquarters, the best men would get the best places, and no one would have to linger on in a subordinate position, hoping for promotion until he was too old for promotion, and until he was too settled in his mode of life to take up any other. An additional advantage of the change would be that the Asylum Service would become a charge upon the taxes instead of a charge upon the rates, a change which economists of high repute have considered just.

No change in any social institution is wholly advantageous, and a disadvantage of converting the Asylum Service into a State Service might be that what research is now conducted would be discountenanced and arrested. It is difficult to imagine scientific research conducted by officials of the State, under the orders of a central office. There has, however, been of late such a clamorous assertion of the importance of scientific research that it must have reached the ears even of Government officials; and it should be the task of the medical profession when the Bill is going through Parliament to insist upon provision for the continuance of such excellent work as is being carried on in the London County Laboratory at Claybury.

